# Chitosan-Stabilized Silver–Nimesulide Coordination Complex with Enhanced Anti-Inflammatory and Redox-Modulating Activity

**DOI:** 10.1021/acsomega.5c09677

**Published:** 2026-03-24

**Authors:** Luis Eduardo M Narvaez, Kely C. N. Lima, Roseane G. Ferreira, Silvia Letícia de F. Gaspar, Victor Ricardo C. Sousa, Rayane Caroline dos S Pereira, Jaqueline D. N. Martins, Lucas da S. Gato, Sávio M. dos Santos, Lisa Maria M. de A. Souza, Carla Carolina F. Meneses, Paulo Robson M. de Sousa, Cláudio N. Alves, Marta C. Monteiro

**Affiliations:** † Health Science Institute, 37871 Federal University of Pará, Belém 66075900, Brazil; ‡ Laboratory Immunology, Microbiology and *In vitro* Assays (LABEIM), Faculty of Pharmacy, Health Science Institute, Federal University of Pará, Belém 66075-110, Brazil; § Pharmaceutical Science Post-Graduation Program, Faculty of Pharmacy, Health Science Institute, Federal University of Pará, Belém 66075900, Brazil; ∥ School of Pharmacy, Health Science Institute, Federal University of Pará, Belém 66075110, Brazil; ⊥ Neuroscience and Cellular Biology Post Graduation Program, Institute of Biological Sciences, Federal University of Pará, Belém 66075110, Brazil; # Laboratory of Drug Planning and Development, Institute of Exact and Natural Sciences, Federal University of Pará, Belem 66075110, Pará, Brazil

## Abstract

Inflammation and oxidative stress are central mediators of acute and chronic diseases. Here, we present a chitosan-stabilized silver–nimesulide coordination complex engineered to enhance anti-inflammatory and redox-modulating performance while enabling controlled drug release. Spectroscopic and crystallographic analyses confirmed the formation of an Ag–NMS coordination core embedded within a polymeric chitosan matrix. The biocomposite significantly reduced carrageenan-induced edema, MPO activity, and lipid peroxidation, while modulating thiol-dependent redox responses. These findings demonstrate that coordination-driven polymeric stabilization improves the therapeutic profile of nimesulide and supports further preclinical investigation of this multifunctional biocomposite.

## Graphical abstract

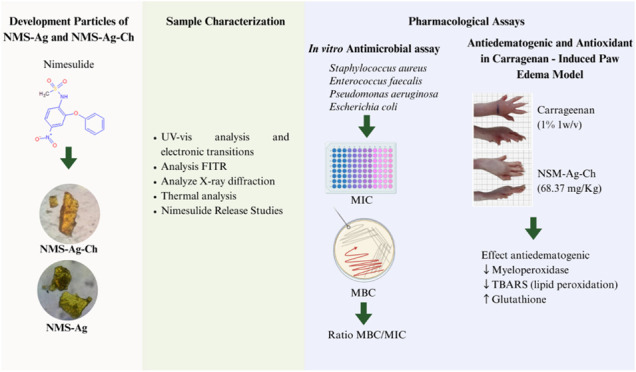

## 1 Introduction

Inflammation is a protective physiological response designed to support tissue repair and promote the neutralization and elimination of pathogens and cellular debris through the release of pro-inflammatory cytokines and the activation of vascular reconstruction pathways at the affected site. However, when inflammation becomes persistent, it may shift from a beneficial to a detrimental process, resulting in chronic inflammation characterized by tissue degradation and oxidative damage. Chronic inflammatory states are implicated in several bone and connective tissue disorders and contribute to the pathogenesis of multiple chronic and degenerative diseases. Consequently, inflammation continues to be a subject of significant scientific interest, particularly regarding the development of therapeutic strategies, as nonsteroidal anti-inflammatory drugs (NSAIDs) remain the primary pharmacological approach.^1–5^

NSAIDs exert their activity mainly through inhibition of COX-1 and COX-2 enzymes, which catalyze the conversion of arachidonic acid into prostaglandins and thromboxanes. These mediators are essential for gastric mucosal protection, regulation of renal hemodynamics, platelet aggregation, and thermoregulation. Thus, while inhibition of COX enzymes attenuates inflammatory signaling, it also contributes to adverse effects such as gastric lesions, renal impairment, and hepatotoxicity. Prolonged NSAID use has further been associated with enhanced production of reactive oxygen species, reduced antioxidant defenses, and mitochondrial dysfunction, ultimately promoting oxidative stress and cell death.^6–10^ To partially overcome these issues, nimesulide—a preferential COX-2 inhibitor—has been employed as it selectively targets COX-2, which is primarily responsible for pain and inflammation, thereby reducing gastrointestinal side effects.^11^ Nimesulide is a weakly acidic secondary amine widely prescribed for the treatment of mild to moderate pain and chronic inflammatory disorders, including rheumatoid arthritis and bone-related conditions. Nevertheless, despite its improved gastrointestinal safety profile, prolonged use of nimesulide is not devoid of risks.^12,13^

To address these limitations, alternative formulations have been increasingly explored to enhance the therapeutic efficacy and safety of anti-inflammatory agents. One promising strategy involves the development of multifunctional bioactive composites with combined anti-inflammatory and antimicrobial properties, capable of reducing adverse effects while improving therapeutic outcomes.^14^ Silver, a transition metal with well-established antimicrobial and immunomodulatory activities, is often incorporated into such systems due to its ability to stabilize the microenvironment, penetrate cell membranes, suppress pro-inflammatory signaling, modulate immune cell trafficking, and inhibit both bacterial and fungal growth.^15^ Previous studies have demonstrated that silver, when combined with bioactive molecules, enhances both anti-inflammatory and antimicrobial effects, including efficacy against Gram-positive bacteria and *Pseudomonas aeruginosa*.^16,17^ To improve stability and enable controlled release, biopolymers such as chitosan are commonly employed as carriers, thereby reducing systemic toxicity and side effects associated with silver- or drug-based systems.^18,19^

In this context and considering the widespread use yet intrinsic limitations of nimesulide and other NSAIDs—including adverse effects, short duration of action, and dose adjustments required for chronic therapy^15,16^ —this study proposes the development of a polymeric biocomposite comprising nimesulide, silver, and chitosan.^20^ The aim is to improve the anti-inflammatory and antimicrobial performance of nimesulide. To this end, the anti-inflammatory, antioxidant, and antimicrobial properties of the nimesulide–silver–chitosan complex were evaluated through both *in vitro* and in vivo assays, including the carrageenan-induced paw edema model.

## 2 Results and Discussion

### 2.1 Development of NMS-Ag and NMS-Ag-Ch Coordination Systems

In this study, we investigated the synthesis, structural characterization, and drug release behavior of a silver–nimesulide (NMS-Ag) complex and its incorporation into a chitosan matrix. Spectroscopic (ultraviolet–visible (UV–Vis) and Fourier-transform infrared (FTIR)) and X-ray diffraction (XRD) analyses confirmed the formation of coordination bonds between nimesulide and silver ions, as well as the influence of chitosan on molecular organization and release properties. Drug release studies in aqueous medium at physiological pH (7.4) revealed that the NMS-Ag complex followed a diffusion-controlled mechanism. The NMS–Ag–Ch system exhibited a more complex release mechanism involving both polymer swelling and polymer erosion, defined as the gradual mass loss of the polymer matrix via chain scission and solubilization of polymer fragments. This process, combined with matrix relaxation, is consistent with the release profile described by the Korsmeyer–Peppas model. The biological evaluation included antimicrobial activity against Gram-positive (*Staphylococcus aureus* and *Enterococcus faecalis*) and Gram-negative (*Escherichia coli* and *Pseudomonas aeruginosa*) bacteria for NMS, Ag, NMS-Ag, and NMS-Ag-Ch, as well as anti-inflammatory effects assessed using the carrageenan-induced paw edema model. It is important to clarify that the material developed in this study is not a discrete nanoparticulate system. Instead, it constitutes a polymer-stabilized coordination complex in which Ag–NMS units are embedded within a chitosan matrix. As such, typical descriptors for nanoparticulate systems (e.g., hydrodynamic diameter or ζ-potential) are not applicable in the dry, solid-state form studied here. Future studies will include hydrated-state analyses to better characterize colloidal behavior under physiological conditions.

### 2.2 Sample Characterization

#### 2.2.1 UV–Vis Analysis and Electronic Transitions

The absorption spectroscopy is a highly useful method for studying drug-metal complex binding because it monitors changes in a complex’s electronic structure, which can be linked to specific binding events. In this work, UV–Vis spectra of pure (Ch and NMS), Ag–NMS and Ag-NMS-Ch complex samples are investigated from 200 to 700 nm, as shown in Figure 1. The chitosan spectrum displays two absorption bands at 214 nm, resulting from the amide linkages, as it is the only partially deacetylated chitin and at 311 nm attributed to intra ligand n → π and π → π* transitions of the chromophoric C=O group. On the other hand, the NMS spectrum is dominated by π–π* transitions and exhibits an absorption band in the visible region at 389 nm. This transition is centered in the nitro-aryl ring and it is low-lying due to the electron-with drawing properties of the NO_2_ substituent. Other absorption bands in the UV region at 281 and 331 nm. Also, the shift of the band from 389 to 450 nm in the Ag-NMS and Ag-NMS-Ch complex sample indicating the completely complexation of Ch with Ag­(I), which is responsible for the Ag–NMS complex intense yellow color (Figure 1).^22^ Such interactions may stabilize silver particles within the polymeric matrix,^24,25^ possibly through chelation mechanisms, as previously reported.^23,25,26^

**Figure 1:**
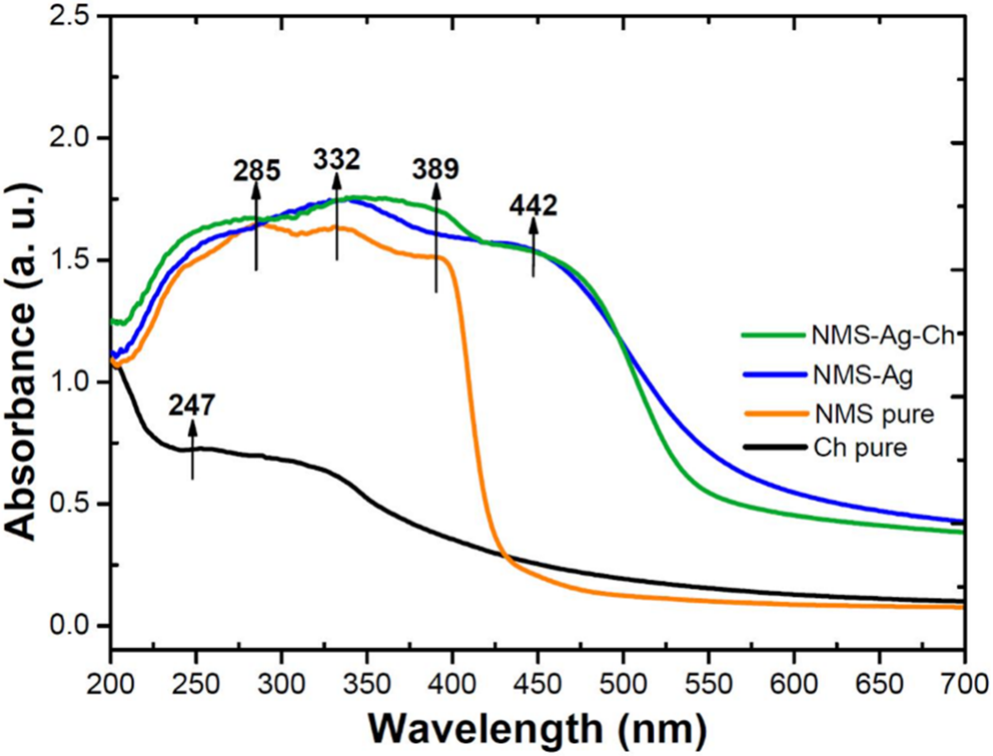
Solid-state UV–vis spectra of the pure Ch (black line), pure NMS (orange line), NMS-Ag (blue line), NMS-Ag-Ch (green line) complex samples.

The NMS-Ag-Ch complex sample exhibited multifunctional proof-of-concept biological activity with controlled release, anti-inflammatory, antimicrobial, and antioxidant effects, making it a system that supports further investigation for biomedical applications.

#### 2.2.2 FTIR Analysis

The infrared spectrum of NMS displayed several characteristic peaks. The N–H stretching vibration appeared as a sharp band at 3282 cm^–1^ (Figure 2A), while the asymmetric and symmetric stretching vibrations of the sulfonyl group (O=S=O, ν_as and ν_s) were observed at 1330 and 1149 cm^–1^, respectively. The nitro group (O=N=O) showed an asymmetric stretching vibration at 1523 cm^–1^ and a symmetric stretching at 1384 cm^–1^. In addition, the C–S stretching band was identified at 756 cm^–1^.^27,28^ However, in the spectrum of the NMS–Ag complex, the N–H band disappeared (Figure 2A), and the symmetric and asymmetric stretching bands of the sulfonyl group shifted to lower wavenumbers, suggesting a weakening of the S=O bond upon coordination with silver. For the nitro group, the asymmetric stretching peak shifted from 1523 to 1495 cm^–1^, while the symmetric stretching band remained at 1384 cm^–1^.

**Figure 2:**
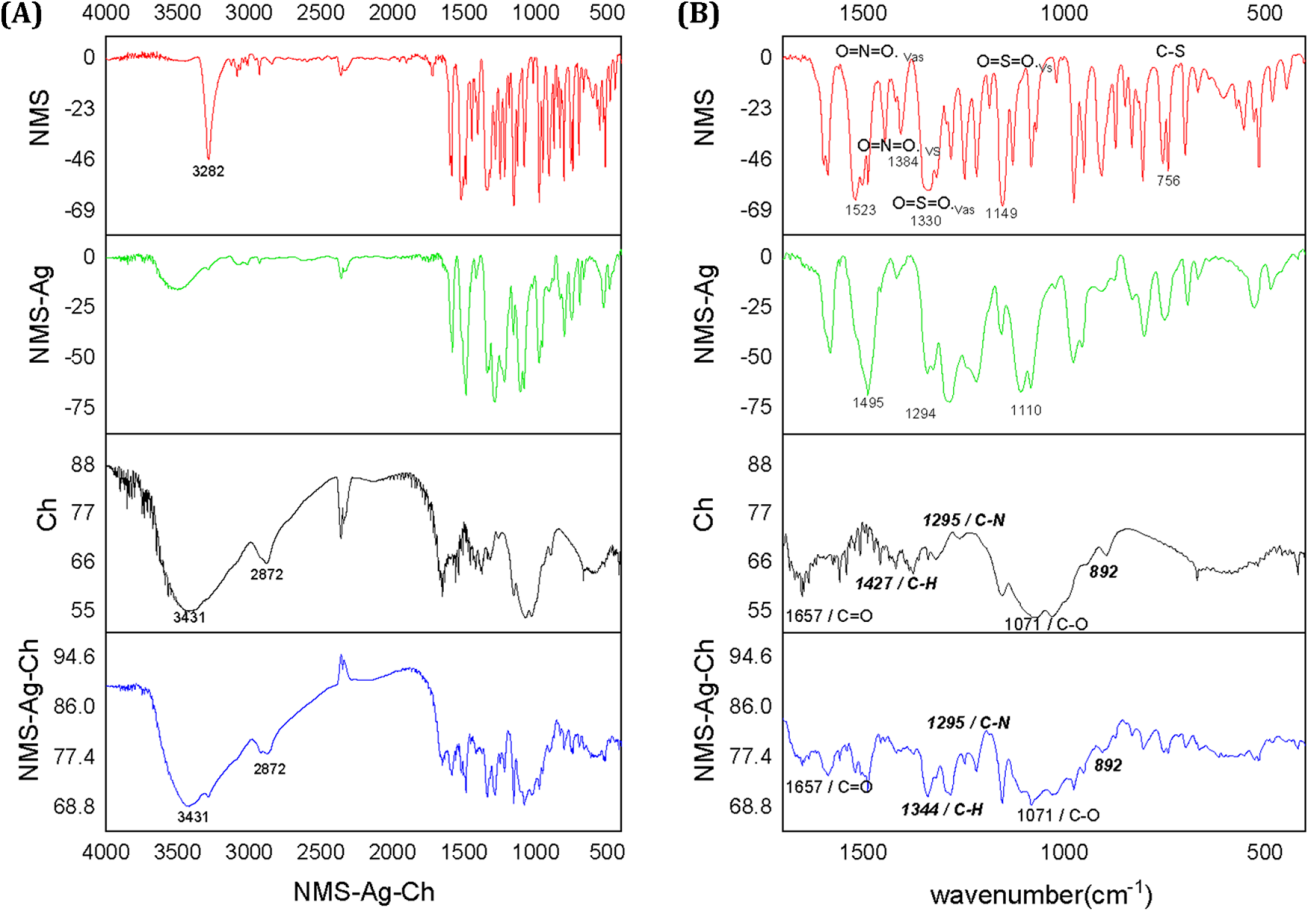
(A) FTIR spectra obtained for the different samples of NMS, NMS-Ag, Ch and NMS-Ag-Ch (B) highlighting the characteristic wavelengths of the functional groups and the observed shifts: NMS (red line), NMS-Ag (green line), chitosan - Ch (black line) and NMS-Ag-Ch (blue line).

The spectra of pure Ch and the polymeric system containing the NMS–Ag complex (NMS–Ag–Ch) were also analyzed. Chitosan exhibited characteristic bands at 3431 cm^–1^ (O–H and N–H stretching), 2872 cm^–1^ (C–H stretching, Figure 2A), 1657 cm^–1^ (C=O stretching of the amide group, – N–C=O), 1427 cm^–1^ (methylene stretching, – CH_2_−), 1322 cm^–1^ (C–N stretching of amino groups), and 892 cm^–1^ (vibrations of the β-glycosidic skeleton). In the spectrum of the NMS–Ag–Ch composite, these bands remained present but displayed intensity variations and slight shifts, particularly at 1657, 1344, 1295, 1071, and 892 cm^–1^ (Figure 2B).

#### 2.2.3 X-ray Diffraction (XRD) Analysis

XRD is a widely applied technique for identifying the crystalline structure of materials, as well as for confirming synthesis and detecting possible structural modifications in newly obtained compounds. In this study, XRD confirmed the formation of the NMS–Ag complex, corroborating the FTIR results. The diffractogram of pure NMS exhibited characteristic peaks consistent with its crystallographic nature (Figure 3), in agreement with previously reported data.^28^ However, these peaks disappeared or underwent significant changes after complex formation, indicating structural rearrangements. The emergence of new reflections suggests the formation of a distinct crystalline phase with different lattice planes, attributable to strong interactions among the components and the establishment of covalent coordination bonds that distort the original crystalline network.

**Figure 3:**
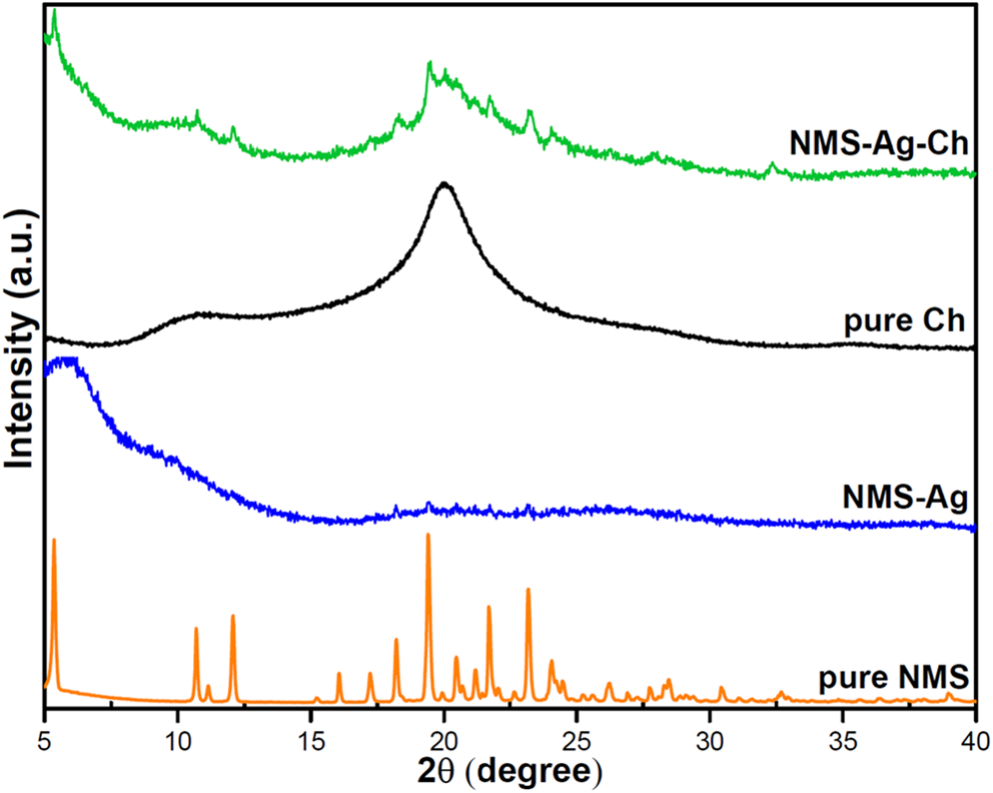
X-ray diffraction patterns of pure NMS, Ag-NMS complex, pure chitosan, and the Ag-NMS-chitosan association. Pure NMS shows well-defined characteristic peaks, indicating high crystallinity. These peaks disappear in the Ag-NMS system, suggesting a loss of structural order after complexation with silver. In the Ag-NMS-chitosan association, some NMS peaks reappear, indicating possible partial release of the molecule, while the overall profile resembles that of chitosan, with slight peak shifts due to interactions among the components.

These diffractometric changes confirm the structural rearrangement associated with Ag­(I) coordination and the embedding of the NMS–Ag complex within the chitosan matrix. Interpretation related to drug-release behavior is presented in Section 2.2.5.

#### 2.2.4 Thermal Analysis

Thermal analysis revealed distinct degradation patterns for the individual components and the composite systems. The NMS–Ag complex exhibited a single thermal event between 235.04 and 343.38 °C, corresponding to a mass loss of 55.98%. The maximum degradation rate occurred at approximately 315.54 °C, followed by complete decomposition of the constituents and leaving a residual mass of 35.53% (Figure 4A).

**Figure 4:**
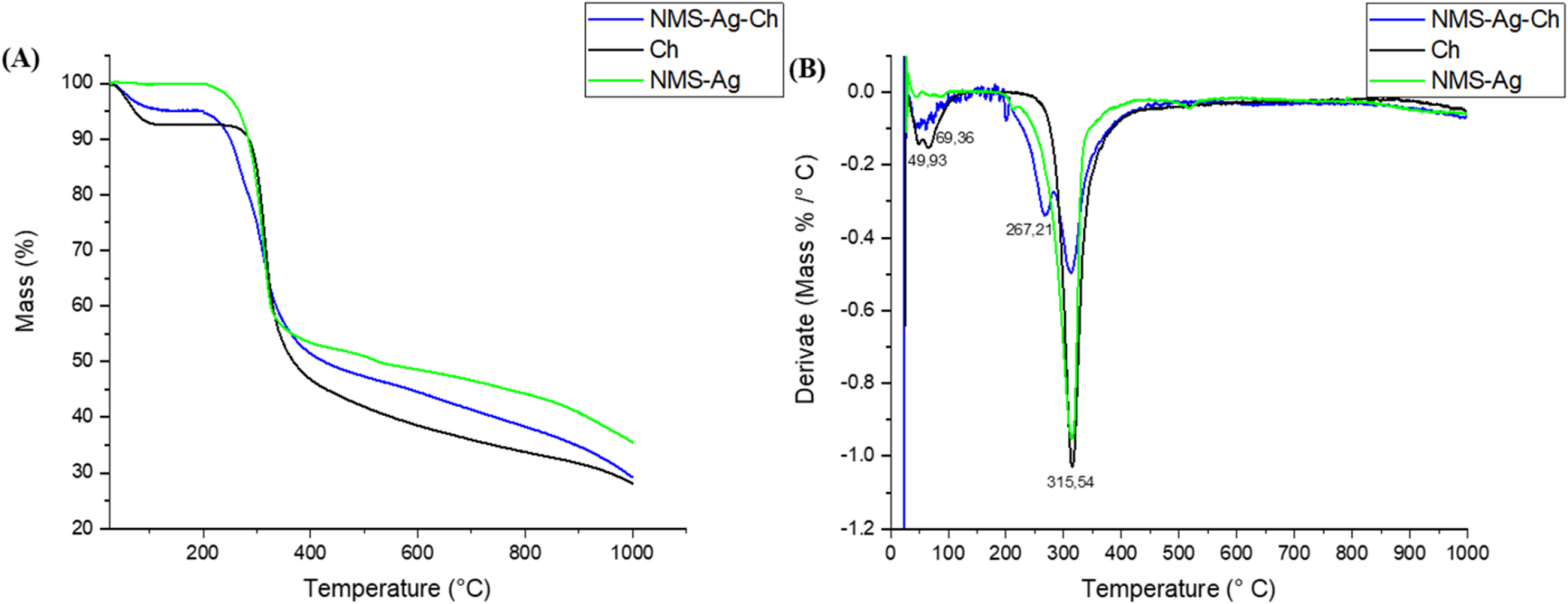
(A) Thermogravimetric graphs from 25 to 1000 °C for each sample, NMS-Ag, Ch and NMS-Ag-Ch. (B) DTG with the identification of each of the mass loss or material degradation events for each of the samples analyzed.

In contrast, chitosan displayed two characteristic thermal events (Table 1). The first, between 34.80 and 109.80 °C, was associated with the loss of adsorbed and capillary water, with two distinct peaks at 49.93 °C (free water) and 69.36 °C (capillary water), consistent with the hygroscopic nature of polymeric materials.^29^ The second event, observed between 266.97 and 355.88 °C, showed the greatest mass loss at 315.54 °C, corresponding to the degradation of chitosan polymer chains.

**Table 1:** Mass Loss Events for the Different Samples Evaluated with the Value of Initial Temperature of the Event (*T*_i_), Final Temperature of the Event (*T*_f_) and Maximum Velocity of Mass Loss (*V*_max_)

sample	event	*T*_i_	*T*_f_	*V*_max_ (%mass/°C)	mass lost (%)	residue (%)
NMS-Ag	1	235.04	343.38	315.54	55.98	44.02
		343.38	998.28		8.49	35.53
Ch	1	34.80	109.80	49.93–69.36	7.93	92.7
	2	266.97	355.88	315.54	41.56	51.14
		355.88	991.9		22.74	28.40
NMS-Ag-Ch	1	38.97	128.67	53.97	5.13	94.87
	2	201.71	387.25	267.21–315.54	42.66	52.21
		387.25	998.28		22.74	29.47

The NMS–Ag–Ch composite also exhibited two thermal events. The first, between 38.97 and 128.67 °C, with a maximum mass loss at 53.97 °C, was attributed to water elimination. The second, spanning 201.71 to 387.25 °C, showed two distinct peaks: the first at 267.21 °C, associated with the release of free NMS, and the second at 315.54 °C, attributed to the degradation of both the NMS–Ag complex and the chitosan polymeric matrix (Figure 4B).

Although thermal analysis does not directly assess biological performance, the thermal stability of the coordination complex and the polymeric matrix are essential for biomedical applications. The defined degradation events indicate that the material remains structurally intact at physiological temperatures, tolerates processing and sterilization conditions, and maintains the integrity required for controlled drug release.

#### 2.2.5 Nimesulide Release Studies

For the NMS–Ag complex, 35.09% of the drug was released within the first 30 min, indicating high stability of the complex under the experimental release conditions. The overall release extended for more than 60 h. In contrast, the NMS–Ag–Ch system released 41.97% of NMS during the first 30 min, reaching a cumulative release of 70.64% within approximately 30 h (Figure 5). These results demonstrate that incorporation into the chitosan matrix accelerates drug release while reducing the overall release time compared with the NMS–Ag complex, underscoring the role of polymeric association in modulating release kinetics.

**Figure 5:**
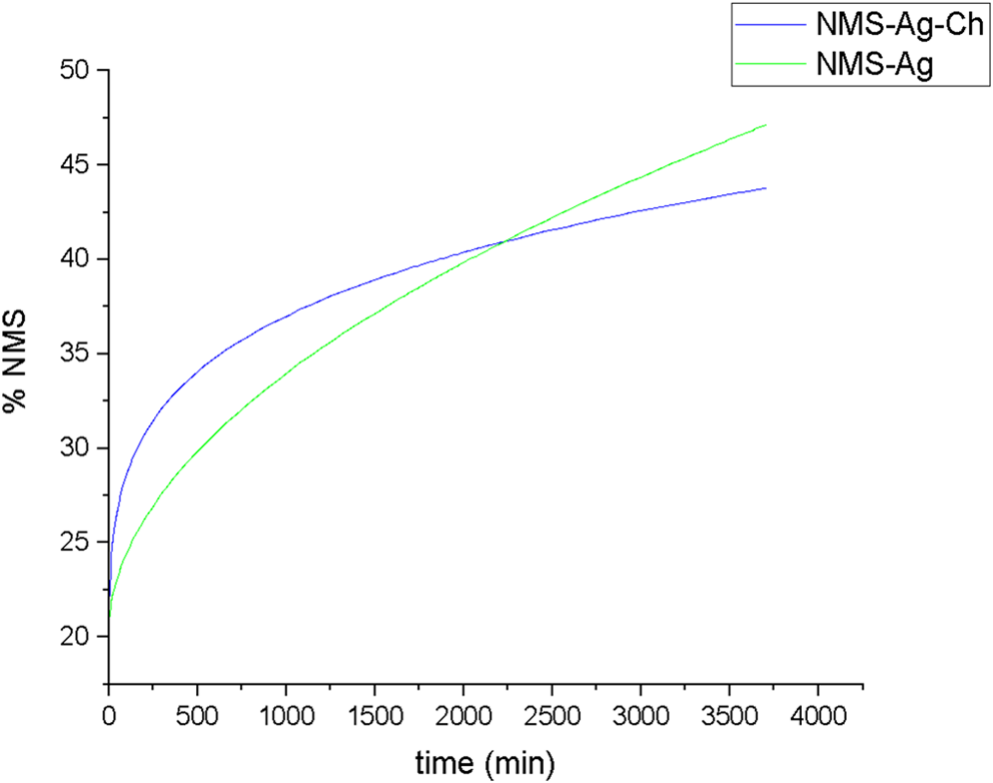
NMS release curve for the NMS-Ag complex and the NMS-Ag-Ch polymeric association.

After processing the raw data, the most suitable kinetic models were identified for each sample. The release profile of the NMS–Ag complex exhibited the best fit to the Higuchi model (Figure 6A), indicating a predominantly diffusion-controlled mechanism. In contrast, the NMS–Ag–Ch system demonstrated a superior fit to the Korsmeyer–Peppas model (Figure 6B), suggesting a more complex release process likely involving both diffusion and relaxation of the polymeric matrix.^30^ These correlations are illustrated in Figure 6 and summarized in Table 2.

**Figure 6:**
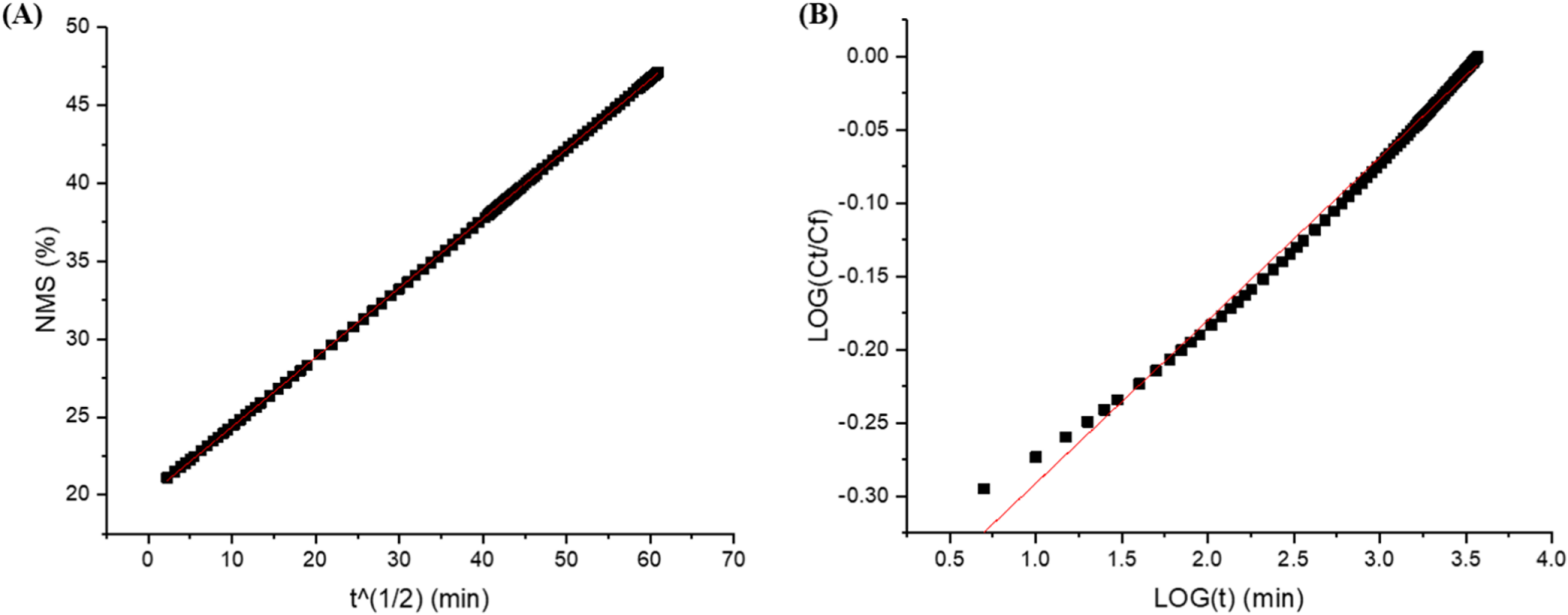
Linearity curve for the NMS-Ag complex and the NMS-Ag-Ch polymeric association using the (A) Higuchi mathematical model and the (B) Korsmeyer-Peppas mathematical model.

**Table 2:** Linearity Values of the Evaluated Models, *a* (Slope), *b* (Intercept) and (*r* ^2^) R-Square for the NMS-Ag Complex and the NMS-AG-Ch Polymeric Association

model name	mathematical equation	linear form	*a*	*b*	*r* ^2^
Higuchi Model	$Q=k_{H}\cdot \sqrt{t}$	$Q\;\mathrm{vs}\;\sqrt{t}$	0.44	19.91	0.999
Korsmeyer–Peppas Model	$\frac{M_{t}}{M_{\infty }}=k\cdot t^{n}$	$\log\left(M_{t}/M_{\infty }\right)=\log k+n\cdot \log t$	0.11	–0.402	0.994

In this regards, the spectroscopic, crystallographic, thermal, and release-kinetic analyses demonstrate that the silver–nimesulide coordination complex remains structurally integrated within the chitosan matrix. These physicochemical features are directly associated with the observed biological performance, as polymer stabilization and controlled release are known to influence drug bioavailability, local concentration, and pharmacodynamic response.

It should be noted that the NMS–Ag–Ch material behaves as a polymer-stabilized coordination complex rather than as a discrete nanoparticulate system. Accordingly, its release profile is governed by polymer–drug interactions and matrix relaxation, rather than nanoparticle-specific properties.

### 2.3 Pharmacological Assays

#### 2.3.1 *In Vitro* Antimicrobial Assay

The antimicrobial activity of NMS, Ag, NMS–Ag, and NMS–Ag–Ch was evaluated against Gram-positive and Gram-negative bacteria, as summarized in Tables 3 and 4. As expected, isolated NMS did not exhibit antibacterial activity at the tested concentrations (≥0.5 mg/mL), consistent with its classification as a nonsteroidal anti-inflammatory drug (NSAID). In contrast, Ag and NMS–Ag demonstrated significant antimicrobial activity against both Gram-positive (*S. aureus* and *E. faecalis*) and Gram-negative (*E. coli* and *P. aeruginosa*) strains, indicating that the incorporation of silver and its complexation with nimesulide conferred relevant bactericidal properties. Among the tested groups, Ag alone exhibited the lowest MIC and MBC values, particularly against *E. faecalis* and the Gram-negative strains, which were the most susceptible.^31,32^ This finding highlights the potent bactericidal activity of silver, widely attributed to its ability to disrupt bacterial cell membranes, interact with DNA, and inactivate essential enzymes, ultimately leading to cell death.^33^ The complexation of nimesulide with silver (NMS–Ag) resulted in a slight increase in MIC and MBC values for both Gram-positive and Gram-negative bacteria compared with silver alone. Nevertheless, NMS–Ag retained a bactericidal effect against all tested strains, demonstrating that, despite the modest reduction in potency, the complex remained effective in inhibiting and killing bacteria (Tables 3 and 4). Regarding the incorporation of chitosan into the system (NMS–Ag–Ch), Table 3 shows that its antimicrobial effects varied depending on the bacterial cell wall. Specifically, NMS–Ag–Ch exhibited a bacteriostatic effect against Gram-positive bacteria, similar to that observed for Ag and NMS–Ag. The antimicrobial profile was classified as bacteriostatic according to the established criterion of an MBC/MIC ratio ≥ 4.^34^ In contrast, the NMS–Ag–Ch system exhibited bactericidal activity against the Gram-negative strains *P. aeruginosa* and *E. coli*.^35–37^ Furthermore, the particulate system displayed lower MIC and MBC values for Gram-negative bacteria compared with Gram-positive strains, suggesting a stronger antimicrobial effect, likely associated with interactions involving the more complex cell wall architecture of Gram-negative bacteria (Table 4).

**Table 3:** Antibacterial Activity of NMS, Ag, NMS–Ag, and NMS–Ag–Ch against Gram-Positive ATCC Strains (*S. aureus* and *E. faecalis*), Expressed as Minimum Inhibitory Concentration (MIC), Minimum Bactericidal Concentration (MBC), and MBC/MIC Ratio (mg/mL^–1^)­^a^

	*S. aureus*			*E. faecalis*		
sample	MIC (mg/mL)	MBC (mg/mL)	MBC/MIC	MIC (mg/mL)	MBC (mg/mL)	MBC/MIC
NMS	ND	ND	ND	ND	ND	ND
Ag	0.031 ± 0.028	0.031 ± 0.000	1 (Bcdl)	0.031 ± 0.005	0.062 ± 0.008	2 (Bcdl)
NMS-Ag	0.031 ± 0.015	0.031 ± 0.031	1 (Bcdl)	0.0625 ± 0.0315	0.125 ± 0.125	2 (Bcdl)
NMS-Ag-Ch	0.0156 ± 0.000	0.125 ± 0.125	8 (Bctc)	0.0625 ± 0.000	0.25 ± 0.000	4 (Bcdl)

a ND: Not determined.

**Table 4:** Antibacterial Activity of NMS, Ag, NMS–Ag, and NMS–Ag–Ch against Gram-Negative ATCC Strains (*E. coli* and *P. aeruginosa*), Expressed as Minimum Inhibitory Concentration (MIC), Minimum Bactericidal Concentration (MBC), and MBC/MIC Ratio (mg/mL^–1^)­^a^

	*E. coli*			*P. aeruginosa*		
sample	MIC (mg/mL)	MBC (mg/mL)	MBC/MIC	MIC (mg/mL)	MBC (mg/mL)	MBC/MIC
NMS	ND	ND	ND	ND	ND	ND
Ag	0.003 ± 0.00012	0.003 ± 0.00020	1 (Bcdl)	0.003 ± 0.000155	0.003 ± 0.00024	1 (Bcdl)
NMS-Ag	0.007 ± 0.00030	0.007 ± 0.00050	1 (Bcdl)	0.007 ± 0.00035	0.007 ± 0.00055	1 (Bcdl)
NMS-Ag-Ch	0.007 ± 0.00050	0.007 ± 0.00080	1 (Bcdl)	0.007 ± 0.00055	0.007 ± 0.00085	1 (Bcdl)

a ND: Not determined.

#### 2.3.2 Antiedematogenic and Antioxidant Effects of NMS–Ag–Ch in the Carrageenan-Induced Paw Edema Model

The carrageenan-induced paw edema model is characterized by a biphasic inflammatory response involving early release of histamine and serotonin, followed by a second phase dominated by COX-2 upregulation, prostaglandin synthesis, neutrophil infiltration, MPO activation, and ROS generation. In our study, carrageenan produced a robust edematogenic response, along with increases in MPO activity, consistent with acute inflammatory activation. Treatment with NMS–Ag–Ch produced the most pronounced reduction in paw edema, surpassing the effects of NMS and NMS–Ag. This superior efficacy is consistent with the multifunctional profile of the composite. Nimesulide contributes COX-2–selective inhibition, while silver species modulate inflammatory responses through suppression of neutrophil recruitment and ROS generation. Chitosan further enhances the anti-inflammatory effect through membrane interactions and immunomodulatory properties. MPO levels were significantly reduced in all treated groups, particularly in NMS–Ag–Ch, indicating decreased neutrophil accumulation at the inflammatory site. This correlates with the observed decrease in edema volume, as MPO is a key enzymatic marker of neutrophil-mediated inflammation (Figure 7).

**Figure 7:**
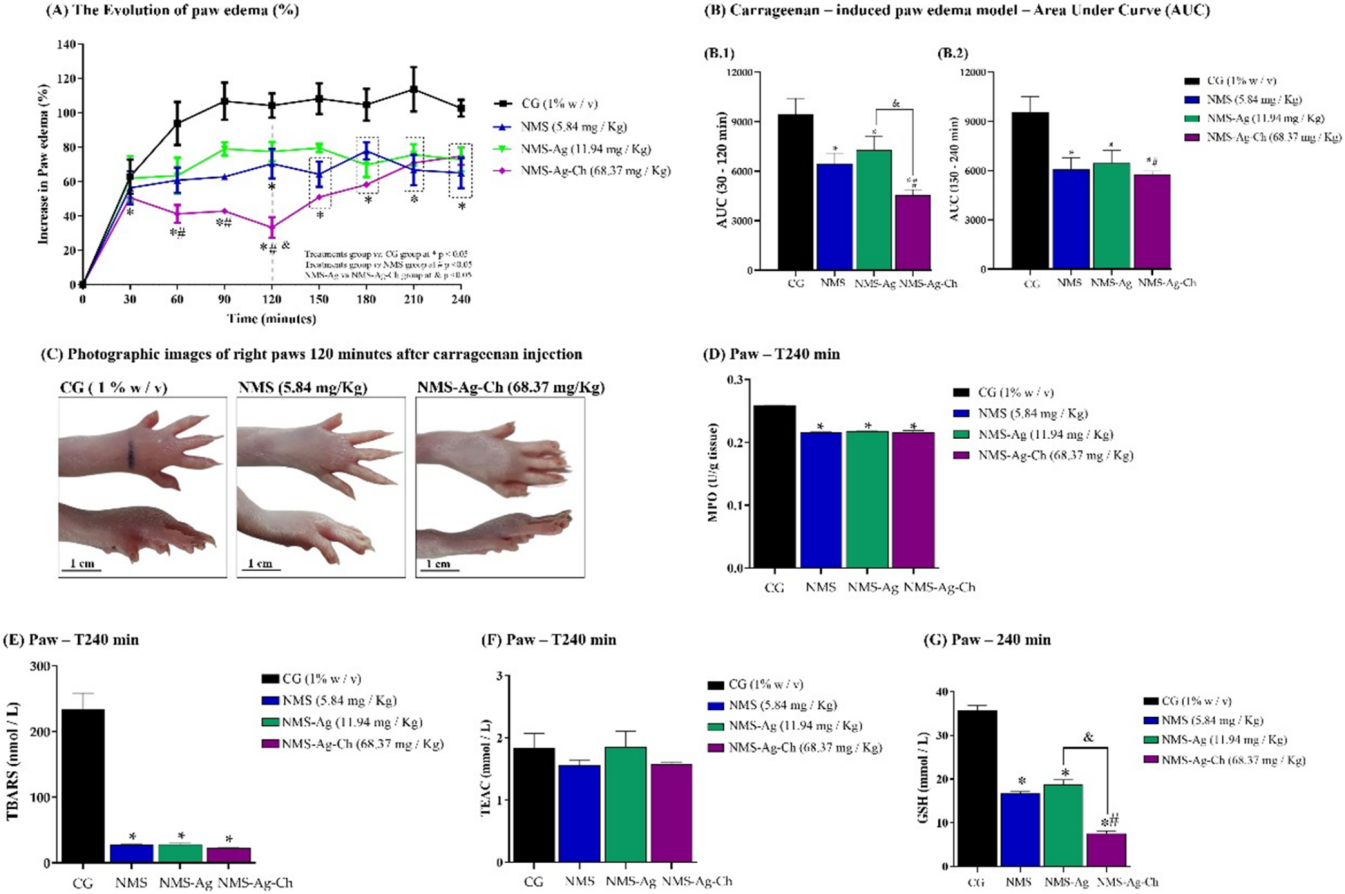
Antiedematogenic and antioxidant effects of NMS (5.84 mg/kg), NMS-Ag (11.19 mg/kg), and NMS-Ag-Ch (68.37 mg/kg) in the carrageenan-induced paw edema model. (A) Time-course of paw edema (% increase from baseline) from 0–240 min. (B1) AUC for the 30–120 min interval. (B2) AUC for the 150–240 min interval. (C) Representative paw photographs at 120 min (scale bar: 1 cm). (D) MPO activity. (E) TBARS levels. (F) TEAC. (G) GSH. Data are mean ± SD (*n* = 5). *p < 0.05 vs CG; #*p* < 0.05 vs NMS; &*p* < 0.05 between NMS-Ag and NMS-Ag-Ch.

Additionally, silver–thiol interactions (including binding to glutathione) may modulate oxidative and inflammatory signaling cascades, such as NF-κB and COX-2 expression, contributing to the overall attenuation of carrageenan-induced injury. The combined effects of the drug, silver coordination, and the polymeric matrix therefore support a synergistic anti-inflammatory action.

Regarding myeloperoxidase (MPO) activity at 240 min, all treated groups showed statistically significant reductions compared with CG (*p* < 0.0001). However, no significant differences were detected among the treatment groups (NMS, NMS–Ag, and NMS–Ag–Ch). Despite the reductions in TBARS and the modulation of GSH levels, TEAC values did not differ significantly across treatments. This finding is consistent with the electron-transfer (ET)-based nature of TEAC, which reflects global antioxidant capacity rather than specific redox pathways. Local modulation of thiol buffering and lipid peroxidation does not always produce detectable changes in ET-based assays. Therefore, the absence of differences in TEAC should not be interpreted as lack of antioxidant activity but rather as a methodological limitation inherent to the assay.

Nimesulide, a preferential COX-2 inhibitor, exerts its anti-inflammatory action by blocking the conversion of arachidonic acid into prostaglandins, particularly PGE_2_, thereby reducing edema formation and cellular infiltration during the late phase of the inflammatory response.^38^ Analysis of the significant reduction in edema volume and leukocyte infiltration supports the conclusion that nimesulide acts predominantly during the second phase of inflammation—a critical stage for assessing compounds with late-phase anti-inflammatory activity. This finding is relevant as it demonstrates the drug’s pharmacodynamic effectiveness in suppressing mediators associated with more complex and persistent inflammatory cascades.^39^

Beyond COX-2 inhibition, increasing evidence suggests that nimesulide exerts pleiotropic effects, including modulation of antioxidant enzyme activity, attenuation of lipid peroxidation, and suppression of pro-inflammatory signaling pathways such as NF-κB.^40^ Importantly, its selectivity for COX-2 is associated with a lower incidence of gastrointestinal adverse effects compared with nonselective NSAIDs, supporting its utility not only as a tool for experimental studies but also as a candidate for clinical applications with an improved safety profile.^41,42^

This study was designed as a proof-of-concept investigation aimed at establishing the synthesis, structural characterization, and initial biological activity of the NMS–Ag–Ch system. Comprehensive cytotoxicity assays, mechanistic studies, and biocompatibility evaluations were beyond the scope of this work and will be conducted in subsequent stages. The biological findings presented here should therefore be interpreted as preliminary and exploratory.

## 3 Conclusion

In conclusion, the chitosan-stabilized silver–nimesulide coordination complex exhibited synergistic structural and pharmacological features, including enhanced anti-inflammatory effects, modulation of redox biomarkers, and strain-dependent antimicrobial activity. The physicochemical characterization confirmed the structural integration of the coordination complex within the polymeric matrix, supporting controlled-release behavior and matrix stabilization. These findings position the NMS–Ag–Ch system as a promising multifunctional platform for future therapeutic applications. Further investigations will focus on cytotoxicity, mechanistic analyses, biocompatibility, and optimization for translational development.

## 4 Experimental Section

### 4.1 Synthesis of the Complex

The synthesis of the complex was adapted from the procedure described by ref 27 with modifications to obtain a pure product. Briefly, 100 mL of an aqueous potassium hydroxide (KOH) solution (8.8 × 10^–3^ mol, 0.4937 g) was reacted with nimesulide (NMS) (8.0 × 10^–3^ mol, 2.4664 g), followed by the addition of 20 mL of an aqueous silver nitrate (AgNO_3_) solution (8.8 × 10^–3^ mol, 1.4948 g). The reaction mixture was stirred continuously at room temperature for 1 h. The resulting yellow solid was recovered by centrifugation at 5000 rpm for 5 min, washed with cold distilled water, and subjected to vacuum filtration. The product was then dried in a desiccator on a heating plate at 40 °C. All steps were performed under light-protected conditions to prevent NMS degradation.

The polymeric association method was adapted from the work of ref 43 with modifications. Chitosan (0.8 g) was dissolved in 100 mL of 1% acetic acid under constant stirring for 1 h. The pH was adjusted to ∼7 by dropwise addition of 1 M sodium hydroxide (NaOH). The NMS–Ag complex (0.4 g) was dispersed in the chitosan solution and stirred for 10 min. The resulting suspension was filtered, repeatedly washed with cold distilled water, and dried at 40 °C.

### 4.2 Sample Characterization

UV–Vis spectroscopy was employed to identify electronic transitions and investigate interactions among NMS, the NMS–Ag complex, and the polymeric NMS–Ag–Ch system. The procedure was adapted from ref 44. Measurements were performed using a Shimadzu UV-2600 spectrophotometer, with samples pressed into the crystal holder. Spectra were recorded between 200–800 nm using barium sulfate as the baseline.

FTIR spectroscopy was used to analyze vibrational changes in functional groups of NMS, particularly amino and sulfonyl groups, and their modifications upon complexation and polymeric association.^21,27^ Analyses were performed on a Shimadzu LANZA spectrometer. Solid samples were finely ground and pressed with KBr to minimize scattering.

Thermogravimetric analysis (TGA) was carried out on NMS–Ag, NMS–Ag–Ch, and pure chitosan to evaluate degradation profiles and potential thermal protection afforded by the polymer. The procedure was adapted from refs 45,46 . Analyses were performed on a Shimadzu TGA-50 in nitrogen (50 mL/min), at a heating rate of 10 °C/min, from 25 to 1000 °C.

Powder X-ray diffraction (XRD) was used to investigate crystalline and amorphous phases. Diffractograms were obtained on a PANalytical X’PERT PRO MPD system using Cu Kα radiation (λ = 1.54184 Å) with a Ni filter, scanning from 5–70° (2θ) at 0.02°/step and 10 s/step.^21^

### 4.3 Nimesulide Release Studies

Drug release kinetics of NMS–Ag and NMS–Ag–Ch were evaluated following protocols adapted from refs 21,47 . Approximately 4 ± 0.02 mg of each polymeric sample was suspended in 2 mL of phosphate buffer (pH 7.4) and incubated at 37 °C with constant agitation.^48–52^ At predetermined time points, aliquots were collected and analyzed at 394 nm using a UV–Vis spectrophotometer, with equivalent volumes of fresh buffer added to maintain sink conditions.

### 4.4 Antimicrobial Activity

Antibacterial activity of NMS, Ag, NMS–Ag, and NMS–Ag–Ch was evaluated against *S. aureus* (ATCC 6538), *E. faecalis* (ATCC 29212), *P. aeruginosa* (ATCC 9027), and *E. coli* (ATCC 8789). Reference strains were provided by INCQS/FIOCRUZ (Rio de Janeiro, Brazil). Bacteria were cultured on Mueller–Hinton agar (Merck, Germany) at 37 °C for 24 h.

#### 4.4.1 Inoculum Preparation

Bacterial suspensions were prepared by transferring 3–4 colonies into 5 mL Mueller–Hinton broth and adjusting the turbidity to 1 × 10^8^ CFU/mL, equivalent to 0.5 McFarland (0.09–0.11 absorbance). Cultures were incubated for 1 h to reach exponential phase, followed by serial dilution to obtain 1 × 10^3^ CFU/mL.^61^

#### 4.4.2 Minimum Inhibitory Concentration (MIC)

MIC was determined by the microdilution method in 96-well plates. Each well contained 100 μL of inoculum and 100 μL of compound solution. Positive controls were chloramphenicol (250 μg/mL, Ariston) for Gram-positive and penicillin/streptolysin (10,000 U/10 mg, Ariston) for Gram-negative strains. Negative controls were inoculum plus 10% DMSO or broth alone. Plates were incubated at 35 °C for 24 h. Experiments were performed in duplicate.^61^

#### 4.4.3 Minimum Bactericidal Concentration (MBC)

MBC was determined by plating 10 μL aliquots from wells onto Mueller–Hinton agar and incubating at 35 °C for 24 h. MBC was defined as the lowest concentration producing no visible colonies or fewer than three colonies, corresponding to a 99.9% reduction in viable cells.^62^

### 4.5 Animal Model

#### 4.5.1 Ethics Statement and Animals

Experiments were conducted in accordance with the Brazilian National Council for Animal Experimentation Control (CONCEA)^3,63^ and national legislation on the scientific use of animals.^64^ Protocols were approved by the Animal Care and Use Committee of the Federal University of Pará (CEUA, Protocol 9782270619). Male Wistar rats (150–200 g, *n* = 20) were obtained from the UFPA animal facility and housed under controlled conditions (25 ± 1 °C, 12 h light/dark cycle, food and water ad libitum). After a 3-day acclimatization, rats were randomly divided into five groups (*n* = 5 each).

#### 4.5.2 Paw Edema Model

Paw edema was induced by subplantar injection of 100 μL carrageenan (1% w/v) in the right hind paw and saline in the left paw, as described previously.^65,66^ Treatments (saline, NMS, NMS–Ag, or NMS–Ag–Ch) were administered into the right thigh (250 μL) at the indicated doses. Paw volume was measured with a digital caliper (Mitutoyo, Japan) at baseline and every 30 min for 240 min. The increase in paw volume was calculated by subtracting the initial paw thickness (*V*_0_) to the paw volume measured at each time point (*V*_f_). Edema (%) was calculated as

$$\%e\mathrm{dema}=\left(V_{f}-V_{0}\right)/V_{0}\times 100)$$

AUC values were determined using the trapezoidal rule.^67,68^

#### 4.5.3 Experimental Design

Animals were assigned to five groups (*n* = 5 each):

- **Carrageenan control:** carrageenan + saline (250 μL, s.c.)
- **NMS + carrageenan:** carrageenan + NMS (5.84 mg/kg, 250 μL)
- **NMS–Ag:** carrageenan + NMS–Ag (11.194 mg/kg, 250 μL)
- **NMS–Ag–Ch:** carrageenan + NMS–Ag–Ch (68.37 mg/kg, 250 μL)

At study completion, animals were anesthetized with ketamine (300 mg/kg) and xylazine (30 mg/kg) and euthanized by cardiac puncture exsanguination.^69^ Paws were collected for biochemical analyses.

#### 4.5.4 Antioxidant Activity

- **MPO Assay:** MPO activity was determined using H_2_O_2_ and TMB as substrates, following the method of ref 53. Absorbance was read at 450 nm.
- **TBARS Assay:** Lipid peroxidation was quantified by TBARS assay, based on the reaction of MDA with thiobarbituric acid.^54–56^ Absorbance was read at 535 nm.
- **TEAC Assay:** Total antioxidant capacity was assessed by the TEAC method.^57,58^ Trolox was used as the standard, and absorbance was read at 734 nm.
- **GSH Assay:** Reduced glutathione was quantified using DTNB to form TNB.^59,60^ Absorbance was read at 412 nm.

### 4.6 Statistical Analysis

Data were analyzed for outliers using the interquartile range method. Student’s *t*-test was used for pairwise comparisons, and ANOVA followed by Tukey’s test was applied for multiple comparisons. Pearson’s correlation was employed to assess associations between variables. Results were considered statistically significant at *p* ≤ 0.05.

## Abbreviations

AUC: area under the curve
CEUA: Animal Ethics Committee
CG: control group
COX-1: cyclooxygenase-1
COX-2: cyclooxygenase-2
CONCEA: National Council for the Control of Animal Experimentation
DNA: DNA
DTNB: 5,5′-dithiobis­(2-nitrobenzoic acid)
DTG: dithioglycol
EDTA: ethylenediaminetetraacetic acid
GSH: reduced glutathione
GlcNAc: *N*-acetylglucosamine
GlcN: glucosamine
N–H: amino group
NMS: nimesulide
NMS–Ag: nimesulide–silver complex
NMS–Ag–Ch: nimesulide–silver–chitosan polymeric association
NSAIDs: nonsteroidal anti-inflammatory drugs
PBS: phosphate-buffered saline
pH: potential of hydrogen
MPO: myeloperoxidase
TBARS: thiobarbituric acid reactive substances
TEAC: Trolox equivalent antioxidant capacity
TLC: thin-layer chromatography
TGA: thermogravimetric analysis
